# Social Media, Depressive Symptoms and Well-Being in Early Adolescence. The Moderating Role of Emotional Self-Efficacy and Gender

**DOI:** 10.3389/fpsyg.2021.660740

**Published:** 2021-05-24

**Authors:** Emanuela Calandri, Federica Graziano, Luca Rollé

**Affiliations:** Department of Psychology, University of Turin, Turin, Italy

**Keywords:** early adolescence, social media, depressive symptoms, well-being, emotional self-efficacy, gender differences, longitudinal

## Abstract

The study of the psychological effects of social media use on adolescents’ adjustment has long been the focus of psychological research, but results are still inconclusive. In particular, there is a lack of research on the positive and negative developmental outcomes and on possible moderating variables, especially concerning early adolescence. To fill these gaps in literature, the present study longitudinally investigated the relationships between social media use, depressive symptoms, affective well-being and life satisfaction, as well as the moderating role of emotional self-efficacy and gender. The study involved 336 Italian early adolescents (mean age = 13, *sd* = 0.3; 48% girls) who completed an anonymous self-report questionnaire twice within a year. Main results showed that higher social media use was related to higher depressive symptoms, lower affective well-being and lower life satisfaction among girls with lower emotional self-efficacy. Conversely, high social media use was related to higher affective well-being and higher life satisfaction for girls with higher emotional self-efficacy. Results are discussed in relation to their implications for risk prevention and health promotion among early adolescents. In particular, our results suggest that promoting emotional self-efficacy can be very helpful in making the use of social media an opportunity for well-being and life satisfaction rather than a developmental risk.

## Introduction

The use of social media has grown exponentially in recent years. Young people are starting their lives on social networks at an increasingly early age, often before the legal age for accessing social networking sites (13 years of age is the minimum age required by most social media services to access and use their services) (Mascheroni and Ólafsson, 2018; IARD, 2019). The use of diverse social media is largely widespread among adolescents and it has become an integral part of their life (OFCOM, 2019). Social media are very attractive to early adolescents and adolescents because they fulfill some of the main developmental tasks of this age, primarily the need for social interaction with peers (Davis, 2012, 2013; Schwartz, 2012) and identity management, showing the positive aspects of oneself and hiding the negative ones (Uhls et al., 2017). Through social media, they can consolidate their sense of belonging to the peer group and defining their identity through a self-disclosure process that is facilitated by the mediation of the device, as well as by the asynchronicity and accessibility of online communication (Donoso and Ribbens, 2010; Valkenburg and Peter, 2011).

Social media are mostly used by teenagers to communicate with their friends, and text messaging has now become the main way that adolescents use to connect with their friends, surpassing face-to-face contact and voice calling (Lenhart, 2015). Some studies highlighted that online communication negatively affects adolescents’ quality of existing friendships because it displaces the time that they could spend into “real” face-to-face interactions with friends (Valkenburg and Peter, 2007). On the contrary, other studies found that in most cases social media encourages communication with existing friends. Therefore, online communication gives adolescents added opportunities to maintain and deepen existing friendships and to increase feelings of closeness to friends (Valkenburg and Peter, 2007; Davis, 2013). Social media use can have a positive effect on friendships provided certain conditions are met; in particular, the communication should involve known friends, not strangers, the activities on social media must have goals that go beyond mere entertainment and recreational purposes, and adolescents should be successful at integrating online disclosure into offline peer interactions (Davis, 2012, 2013).

While most studies refer to adolescents, the role of social media on early adolescents’ friendships has been rarely investigated in literature. The few existing studies found that the use of social network sites was related to higher well-being in peer relationships (Quinn and Oldmeadow, 2013; Ma, 2018), whereas early adolescents who were not members of a social network or used less frequently the social media with friends reported lower friendship quality (Antheunis et al., 2012; Foerster and Röösli, 2017). Other studies focusing on early adolescence, however, seem to confirm a negative impact of social media on mental health. In particular, high social networking use was related to higher depression (Vannucci and McCauley Ohannessian, 2019), especially among early adolescent girls (Mundy et al., 2020).

The debate about the possible negative and positive effects of social media use on young people’s adjustment is still ongoing and current research is characterized by some shortcomings that might be addressed (Schønning et al., 2020). First of all, most existing studies on social media and adolescents’ mental health have cross-sectional designs that limit inference about causality. As outlined in many recent reviews, there is a need of longitudinal studies examining the effect of social media on developmental outcomes over time (Keles et al., 2020; Schønning et al., 2020; Vidal et al., 2020). Some recent studies found a lack of association between the use of social media and subsequent depressive symptoms (Coyne et al., 2020), but research specifically focusing on early adolescence is still lacking. For this reason, our study was based on a longitudinal research design, specifically focused on 1-year span, when participants were aged between 12 and 14 years. This is a crucial time frame because it is the age at which there is an exponential growth in the use of social media (IARD, 2019; OFCOM, 2019), but also an increasing vulnerability to increasing depressive symptoms (McLaughlin and King, 2015).

A second gap in the literature on social media and adolescents’ mental health is the paucity of studies examining the relationship between social media use and positive indicators of mental health, such as well-being (Longobardi et al., 2020; Schønning et al., 2020). For this reason, in the present study, we focused not only on depressive symptoms, but also on the subjective well-being, defined as the individual cognitive and affective positive evaluation of his/her life (Diener et al., 2002). The subjective well-being is the sum of two components: the affective one, defined in terms of hedonic balance, that is the predominance of positive moods and emotions over negative ones, and the cognitive one, defined in terms of life satisfaction (Diener, 1994; Ryan and Deci, 2001; Deci and Ryan, 2008).

In addition to the lack of longitudinal studies and of research considering positive outcomes, a third shortcoming of the research on social media and adjustment in adolescence is the scarce consideration of the effects of potential mediating or moderating factors (Ivie et al., 2020; Orben et al., 2020). In light of the complexity of the topic, it is important to examine the role of intervening variables because this analysis could shed light on the mechanisms of the association between social media use and both positive and negative outcomes (Schønning et al., 2020; Vidal et al., 2020). For this reason, our study focused on emotional self-efficacy that is the perceived ability to regulate and express positive and negative emotions (Bandura et al., 2003). Early adolescence is characterized by great emotional instability and it is a crucial period for the acquisition of the ability of managing and self-regulating emotions (Grazzani et al., 2015; Calandri et al., 2021). Research targeting adolescent samples found that girls generally reported lower emotional self-efficacy than boys, especially in managing negative emotions (Bandura et al., 2003; Grazzani et al., 2015). Low emotional self-efficacy has proven to be related to lower subjective well-being (Bandura, 1997; Caprara and Steca, 2005) and higher depressive symptoms among early adolescents and adolescents, especially girls (Bandura et al., 2003; Calandri et al., 2021). The links between emotional self-efficacy, social media use and mental health in early adolescence is largely unexplored. Social media are an arena for young people to express a wide range of emotions and the communication through social networks has been defined as an “emotional experience”, characterized by both positive and negative affect (Vermeulen et al., 2018; Weinstein, 2018). Social media are used to express and share emotions, to receive feedback from others, as well as to enhance mood, sometimes to impulsively vent negative emotions and find a feeling of relief (Vermeulen et al., 2018). It is plausible that, just as in offline relationships, the ability to manage emotions also plays an adaptive role in online ones. The role of emotion regulation has been generally studied in relation to the problematic use of the Internet in adolescence and studies showed that high levels of social and emotional competencies were related to less technology abuse (Nasaescu et al., 2018) and the ability to regulate and managing emotions can contrast a problematic use of the Internet among adolescents (Bruno et al., 2014; Pace et al., 2019). Nonetheless, these studies did not specifically consider the use of social media, nor were focused on early adolescents. To fill this gap in the literature, we focused on the relationships between social media use, emotional self-efficacy and developmental outcomes (depressive symptoms, affective well-being and life satisfaction) among early adolescents.

A final shortcoming of existing studies is the lack of consideration of possible gender differences in the association between social media use and developmental outcomes, especially for early adolescents (Schønning et al., 2020). Social media use has different characteristics among boys and girls. Boys are more likely to use social media for instrumental exchanges, such as to make plans to see friends (Davis, 2012) or to participate in online gaming (OFCOM, 2019). On the contrary, girls usually give more importance to the emotional aspects of friendships and communication – they spend more time than boys surfing social media and use them especially for emotional self-disclosure (Pujazon-Zazik and Park, 2010; Liang et al., 2016). The different use of social media seems to emerge from early adolescence with characteristics that may be potentially risky for well-being. Previous research obtained mixed results, even though girls seem to be more vulnerable to the detrimental effects of social media use. In particular, the few studies targeting early adolescents showed that high levels of social media interaction in early adolescence had negative implications for later well-being, especially for girls (Mcdool et al., 2016; Booker et al., 2018; Mundy et al., 2020). Considering gender issues might allow a deeper understanding of the complex phenomenon under investigation.

### The Present Study

To sum up, results of the research on the links between social media use and developmental outcomes are still conflicting and most studies considered adolescence, whereas literature on early adolescents (12–14 years old) is still scarce. It is important to consider this period of life because the use of social media is growing at this age, but also mental health problems are emerging, especially depressive symptoms, while individual competencies, like emotional self-efficacy, are still developing (Grazzani et al., 2015; McLaughlin and King, 2015). Moreover, due attention should also be paid to consider positive developmental outcomes, like the subjective well-being, and to longitudinally examine the role of social media on subsequent outcomes, also considering the role of intervening variables (Schønning et al., 2020). In the present study, the emotional self-efficacy was considered as a moderator in the relationships between social media use and subsequent depressive symptoms, affective well-being, and life satisfaction. In other words, we expected that a higher involvement in social media use could have detrimental or positive effects on early adolescents’ depressive symptoms, affective well-being and life satisfaction in relation to their perceived ability of managing emotions. Last but not least, a special attention was paid to gender differences, in light of literature suggesting gender differences in the use of social media (Pujazon-Zazik and Park, 2010; Liang et al., 2016; HBSC (Health Behaviour in School-aged Children), 2018) and in the effects of social media on developmental outcomes (Mcdool et al., 2016; Booker et al., 2018; Mundy et al., 2020). Gender was therefore considered as a possible second moderator in the relationships between social media use, emotional self-efficacy and examined outcomes.

**FIGURE 1:**
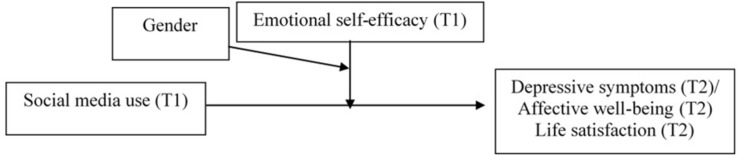
The hypothesized three-way interaction model.

The aims of the study were the following:

(1) To describe the usage rate of social media and levels of depressive symptoms, affective well-being, life satisfaction, and emotional self-efficacy in a group of early adolescents, taking into account gender differences. Consistently with the results of previous studies, we expected that girls reported higher involvement in social media use than boys (Pujazon-Zazik and Park, 2010; Liang et al., 2016). Moreover, girls were expected to report higher depressive symptoms (McLaughlin and King, 2015), lower well-being (González-Carrasco et al., 2017), and lower emotional self-efficacy (Bandura et al., 2003; Grazzani et al., 2015) than boys.
(2) To investigate the relationships between social media use (T1), and subsequent depressive symptoms (T2), affective well-being (T2), and life-satisfaction (T2), and the potential moderating role of emotional self-efficacy (T1). Higher social media use was expected to be related to higher depressive symptoms, lower affective well-being and lower life-satisfaction when levels of emotional self-efficacy were lower.
(3) To investigate if gender further moderates the relationships between social media use, emotional self-efficacy and outcomes (depressive symptoms, affective well-being, and life satisfaction). This analysis was exploratory in nature and no specific hypothesis was formulated (Figure 1).

## Materials and Methods

### Procedure and Participants

A convenience sample of seven middle schools^1^ located in the northwest of Italy was selected to participate in the study. The research project was presented to each school and 26 classes were enrolled. The study was approved by the Bioethics Committee of the University of Turin (Italy), and written informed consent was obtained from the parents of the participants before the questionnaire was administered. Parental consent was given for 96% of the students contacted to participate in the study. Participants completed an anonymous self-report questionnaire, administered by trained researchers in the schools during classroom time, without teachers present. Completed questionnaires were turned in immediately to the researchers. Students were requested to write a self-generated code to combine questionnaires of the two waves. Participants did not receive benefits for participating in the study.

The first wave of data collection (T1) involved 398 early adolescents aged between 12 and 14 years attending the second year of middle school. The second wave of data collection (T2) took place 1 year apart, when students were attending the third year of middle school. A total of 336 students were present at both waves. The attrition rate was 15.6%. Only participants who completed the two assessments were included in the present study (*N* = 161, 48% girls; *N* = 175, 52% boys; age range T1 12–14 years; mean age T1 = 13, *sd* = 0.3; age range T2 13–15 years; mean age T2 = 14, *sd* = 0.3)^2^. Participants included in the study did not differ from the overall sample on demographic or study variables. The majority of participants (85%) lived with both parents and had brothers or sisters (85%). Parents’ level of education was medium-high (high school diploma for 36.1% of mothers and 32.1% of fathers; degree for 25.9% of mothers and 18.4% of fathers). The majority of parents were employed full time (56% of mothers and 82.7% of fathers)^3^.

### Measures

*Social media use*: participants were asked how many hours they spend per day communicating online with friends through social networks (e.g., Instagram and WhatsApp), using a 4-point scale from 0 to 3 (0 = 0 h, 1 = about 1 h, 2 = about 2 h, 3 = 3 h or more).

*Emotional self-efficacy.* Students completed the Multidimensional Negative Regulatory Emotional Self-Efficacy Scale (Caprara et al., 2013) and the Positive Regulatory Emotional Self-Efficacy Scale (Caprara et al., 2008). The 15 items of the first scale evaluated the perceived ability to regulate negative affect (anger, sadness, fear, shame, and guilt), whereas the second includes 4 items regarding the perceived ability to express positive affect. For both scales, participants responded on 5-point Likert scale ranging from 1 (not able at all) to 5 (very able). The total score of the two scales (19 items) ranges from 19 to 95 (Cronbach’s alpha = 0.80).

*Depressive symptoms*. Participants completed the Italian validation of the Center for Epidemiological Studies Scale—short version 10 items (CESD-10) (Pierfederici et al., 1982). The scale evaluates the frequency of depressive symptoms during the past week on a 4-point Likert scale from 0 (rarely or none of the time) to 3 (most or all of the time) (range 0–30; Cronbach’s alpha = 0.67).

*Affective well-being* was evaluated using the Italian validation of the Positive Affect and Negative Affect Schedule (PANAS; Terracciano et al., 2003). It includes two mood scales: Positive Affect (PA) (10 items) and Negative Affect (NA) (10 items); each item is rated on a 5-point scale (from 1 = never to 5 = always) to indicate the number of times the respondent feels this way (e.g., positive: interested; negative: scared) in their daily living. The measure of affective well-being results from the total PA minus the total NA. The scale ranges from −40 to +50 and Cronbach’s alpha was 0.75.

*Life satisfaction* was evaluated using a modified version of the Brief Multidimensional Students’ Life Satisfaction Scale (BMSLSS; Huebner et al., 2006); it includes six items referring to satisfaction with different life domains (health, friendships, school experience, relationships with classmates, family life, and leisure time activities). Response options ranged from 0 (extremely unsatisfied) to 10 (extremely satisfied). Life satisfaction was the sum of the six items (range 0–60; Cronbach’s alpha = 0.69).

### Statistical Analysis

The percentage of missing data for the study variables was less than 10% and the MCAR (Missing Completely at Random) test (Little, 1988) indicated that missing were not completely at random, thus they were imputed through the Regression procedure. Descriptive analyses included *t* tests for gender differences, Cohen’s *d* as a measure of *t*-test effect size, and Pearson’s bivariate correlations.

Then, according to the aims of the study, the PROCESS SPSS-macro (Hayes, 2017) was used to test the hypothesized three-way interaction models. In the first model, we examined the role of social media use (T1) on subsequent depressive symptoms (T2) moderated by emotional self-efficacy (T1) and gender, controlling for depressive symptoms at T1. In the second model, we examined the role of social media use (T1) on subsequent affective well-being (T2) moderated by emotional self-efficacy (T1) and gender, controlling for affective well-being at T1. In the third model, we examined the role of social media use (T1) on subsequent life satisfaction (T2) moderated by emotional self-efficacy (T1) and gender, controlling for life satisfaction at T1. Continuous variables were mean centered. For each model, the statistical significance of the moderation effect was evaluated through a bootstrapping procedure (95% confidence intervals with 5,000 bootstrap samples). Confidence intervals that do not contain zero indicate a statistically significant effect. To interpret significant interactions, a simple slope analysis was performed testing the relationship between the independent (social media use) and the dependent variables (depressive symptoms, affective well-being, and life satisfaction) at low (mean −1 sd) and high (mean +1 sd) levels of the moderator (emotional self-efficacy) in the two groups (boys vs. girls). All statistical analyses were performed with SPSS Statistics 26.

## Results

### Descriptives

Girls spent more time than boys on daily online communication and reported lower emotional self-efficacy than boys. Girls reported higher depressive symptoms, lower affective well-being, and lower life satisfaction than boys at both waves^4^ (Table 1).

**TABLE 1:** Descriptive statistics of the study variables in the total group of participants and by gender in the two waves (T1 and T2) (*N* = 336).

	Total (*N* = 336)		Girls (*N* = 161)		Boys (*N* = 175)		*t*	*df*	*p*	Cohen’s *d*
	*M*	*ds*	*M*	*ds*	*M*	*ds*				
Social media use T1 (h/day)	1.2	0.9	1.4	0.9	1.0	0.8	3.78	334	0.0001	0.47
Emotional self-efficacy T1	62.9	10.1	60.5	9.9	65.1	9.8	–4.23	334	0.0001	0.47
Depressive symptoms T1	7.2	4.1	8.1	4.3	6.4	3.7	3.9	334	0.0001	0.42
Affective well-being T1	9.4	8.0	7.8	7.9	10.9	7.7	–3.68	334	0.0001	0.40
Life satisfaction T1	51.4	6.6	50.4	7.5	52.3	5.5	–2.58	334	0.010	0.29
Depressive symptoms T2	8.2	5.1	9.5	5.7	6.9	4.0	4.90	334	0.0001	0.53
Affective well-being T2	8.2	9.1	5.2	8.5	11.0	8.7	–6.18	334	0.0001	0.67
Life satisfaction T2	50.4	7.7	49.0	8.7	51.6	6.3	–3.21	334	0.001	0.34

Depressive symptoms increased from T1 to T2 [Hotelling’s trace = 0.047; *F*(1,334) = 15.84; *p* = 0.0001], whereas both affective well-being and life satisfaction decreased [Hotelling’ s trace = 0.024; *F*(1,334) = 8.01; *p* = 0.005; Hotelling’s trace = 0.022; *F*(1,334) = 7.32; *p* = 0.007, respectively]. Moreover, the decrease of affective well-being was stronger for girls than for boys [time × gender Hotelling’s trace = 0.026; *F*(1,334) = 8.82; *p* = 0.003], whereas no gender interaction effects emerged for depressive symptoms, nor for life satisfaction [Hotelling’s trace = 0.009; *F*(1,334) = 3.03; *p* = 0.083; Hotelling’s trace = 0.003; *F*(1,334) = 1.11; *p* = 0.294, respectively].

Also time spent using social media increased from T1 to T2 (from 1.18 to 1.38 h/day) [Hotelling’s trace = 0.049; *F*(1,334) = 16.37; *p* = 0.0001] without gender differences [Hotelling’s trace = 0.001; *F*(1,334) = 0.067; *p* = 0.796], whereas emotional self-efficacy remained stable (mean levels from 62.9 to 62.8) [Hotelling’s trace = 0.001; *F*(1,334) = 0.017; *p* = 0.896] for both boys and girls [Hotelling’s trace = 0.013; *F*(1,334) = 3.93; *p* = 0.060].

Correlation analysis showed that higher social media use (T1) was related to higher depressive symptoms (at both T1 and T2) and to lower life satisfaction at T2. Higher emotional self-efficacy (T1) was related to lower depressive symptoms, higher affective well-being, and higher life satisfaction at both waves^5^ (Table 2).

**TABLE 2:** Bivariate correlations between the study variables.

		1	2	3	4	5	6	7	8
(1)	Social media use T1	–							
(2)	Emotional self-efficacy T1	−0.04	–						
(3)	Depressive symptoms T1	0.16**	−0.39**	–					
(4)	Affective well-being T1	−0.04	0.40**	−0.49**	–				
(5)	Life satisfaction T1	−0.06	0.35**	−0.49**	0.42**	–			
(6)	Depressive symptoms T2	0.15**	−0.30**	0.50**	−0.24**	−0.36**	–		
(7)	Affective well-being T2	−0.08	0.44**	−0.47**	0.53**	0.38**	−0.62**	–	
(8)	Life satisfaction T2	−0.14*	0.25**	−0.40**	0.22**	0.51**	−0.53**	0.50**	–

**p < 0.01

### Moderation Analyses

#### Predictors of Depressive Symptoms

To predict depressive symptoms (T2), social media use was entered in the regression model as independent variable, whereas emotional self-efficacy and gender were entered as first and second moderators, respectively. Depressive symptoms (T1) were entered as a covariate. We tested all 2-way interactions (social media use × emotional self-efficacy, social media use × gender, emotional self-efficacy × gender) and the 3-way interaction (social media use × emotional self-efficacy × gender). Results are showed in Table 3. Significant coefficients were observed for depressive symptoms (T1) and gender, as well as for the two-way interaction social media use × emotional self-efficacy and the three-way interaction social media use × emotional self-efficacy × gender.

**TABLE 3:** Predictors of depressive symptoms (T2) (regression analysis).

	*b*	*se*	*t*	*p*	Bootstrapping CI 95%	
					LL (lower limit)	UL (upper limit)
Intercept	5.41	0.60	8.99	0.0001	4.23	6.59
Depressive symptoms (T1)	0.48	0.06	7.44	0.0001	0.35	0.60
Gender	1.43	0.49	–2.90	0.004	–2.40	–0.46
Social media use	0.24	0.36	0.66	0.509	–0.47	0.94
Emotional self-efficacy	–0.07	0.04	–1.90	0.058	–0.14	0.01
Social media use × emotional self-efficacy	–0.12	0.04	–3.34	0.001	–0.20	–0.05
Social media use × gender	–0.09	0.52	–0.18	0.860	–1.13	0.94
Emotional self-efficacy × gender	0.04	0.05	0.79	0.430	–0.06	0.13
Social media use × emotional self-efficacy × gender	0.13	0.05	2.31	0.021	0.02	0.23

The 3-way interaction term slightly increased the explained variance of the model with a *R*-square change of 0.011, *F*(1,327) = 5.35, *p* = 0.021. The *R*-square of the final model was 0.55, *F*(8,327) = 18.26, *p* = 0.0001.

**FIGURE 2:**
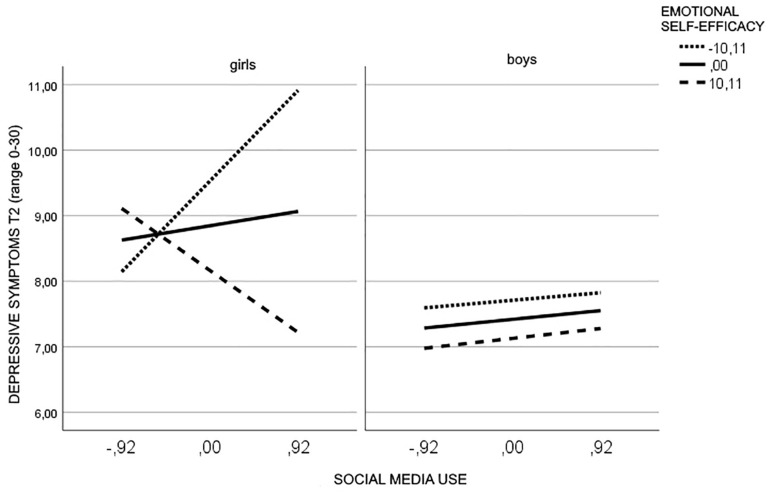
Moderating effect of emotional self-efficacy and gender in the relationship between social media use and depressive symptoms.

The test of simple slopes revealed that the interaction social media use × emotional self-efficacy was significant only for girls (*b* = −0.12, *p* = 0.009). In particular, for girls with low levels of emotional self-efficacy high social media use was positively related to depressive symptoms (*b* = 1.50, *t* = 3.08, *p* = 0.002, 95% CI = 0.54, 2.45) (Figure 2).

#### Predictors of Affective Well-Being

To predict affective well-being (T2), social media use was entered in the regression model as independent variable, whereas emotional self-efficacy and gender were entered as first and second moderators, respectively. Affective well-being (T1) was entered as a covariate. Again, we tested all 2-way interactions (social media use × emotional self-efficacy, social media use × gender, emotional self-efficacy × gender) and the 3-way interaction (social media use × emotional self-efficacy × gender). Results are showed in Table 4. Significant coefficients were observed for affective well-being (T1), gender, and emotional self-efficacy as well as for the two-way interaction social media use × emotional self-efficacy and the three-way interaction social media use × emotional self-efficacy × gender. The 3-way interaction term slightly increased the explained variance of the model with a *R*-square change of 0.009, *F*(1,327) = 4.82, *p* = 0.029. The *R*-square of the final model was 0.40, *F*(8,327) = 27.22, *p* = 0.0001.

**TABLE 4:** Predictors of affective well-being (T2) (regression analysis).

	*b*	*se*	*t*	*p*	Bootstrapping CI 95%	
					LL (lower limit)	UL (upper limit)
Intercept	2.35	0.75	3.13	0.002	0.88	3.83
Affective well-being (T1)	0.44	0.05	8.08	0.0001	0.33	0.54
Gender	3.38	0.82	4.12	0.0001	1.77	5.00
Social media use	–0.14	0.59	–0.24	0.810	–1.31	1.02
Emotional self-efficacy	0.18	0.06	2.93	0.004	0.06	0.29
Social media use × emotional self-efficacy	0.22	0.06	3.56	0.0004	0.10	0.34
Social media use × gender	0.27	0.87	0.31	0.753	–1.44	1.99
Emotional self-efficacy × gender	0.06	0.08	0.74	0.459	–0.10	0.22
Social media use × emotional self-efficacy × gender	–0.20	0.09	–2.19	0.029	–0.38	–0.02

**FIGURE 3:**
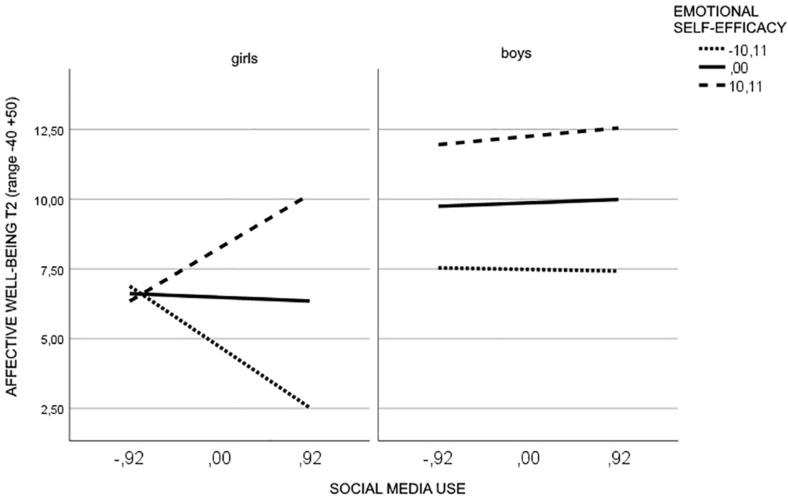
Moderating effect of emotional self-efficacy and gender in the relationship between social media use and affective well-being.

The test of simple slopes showed that the interaction social media use × emotional self-efficacy was significant only for girls (*b* = 0.22, *p* = 0.0004). In particular, for girls with low levels of emotional self-efficacy high social media use was related to lower affective well-being (*b* = −2.36, *t* = −2.98, *p* = 0.003, 95% CI = −3.91, −0.80) whereas for girls with high levels of emotional self-efficacy high social media use was related to higher affective well-being (*b* = 2.07, *t* = 2.25, *p* = 0.025, 95% CI = 0.26, 3.88) (Figure 3).

#### Predictors of Life Satisfaction

To predict life satisfaction (T2), social media use was entered in the regression model as independent variable, whereas emotional self-efficacy and gender were entered as first and second moderators, respectively. Life satisfaction (T1) was entered as a covariate. Again, we tested all 2-way interactions (social media use × emotional self-efficacy, social media use × gender, emotional self-efficacy × gender) and the 3-way interaction (social media use × emotional self-efficacy × gender). Results are showed in Table 5. Significant coefficients were observed for life satisfaction (T1) as well as for the two-way interaction social media use X emotional self-efficacy and the three-way interaction social media use × emotional self-efficacy × gender. The 3-way interaction term slightly increased the explained variance of the model with a R-square change of 0.024, *F*(1,327) = 11.73, *p* = 0.0007. The *R*-square of the final model was 0.33, *F*(8,327) = 20.29, *p* = 0.0001.

**TABLE 5:** Predictors of life satisfaction (T2) (regression analysis).

	*b*	*se*	*t*	*p*	Bootstrapping CI 95%	
					LL (lower limit)	UL (upper limit)
Intercept	22.91	2.95	7.77	0.0001	17.11	28.72
Life satisfaction (T1)	0.52	0.06	9.24	0.0001	0.41	0.64
Gender	1.20	0.73	1.65	0.099	–0.23	2.64
Social media use	–0.80	0.53	–1.51	0.133	–1.83	0.24
Emotional self-efficacy	0.05	0.05	0.93	0.353	–0.05	0.15
Social media use × emotional self-efficacy	0.25	0.06	4.49	0.0001	0.14	0.36
Social media use × gender	0.59	0.78	0.75	0.453	–0.95	2.12
Emotional self-efficacy × gender	–0.04	0.07	–0.50	0.619	–0.18	0.11
Social media use × emotional self-efficacy × gender	–0.28	0.08	–3.42	0.0007	–0.44	–0.12

**FIGURE 4:**
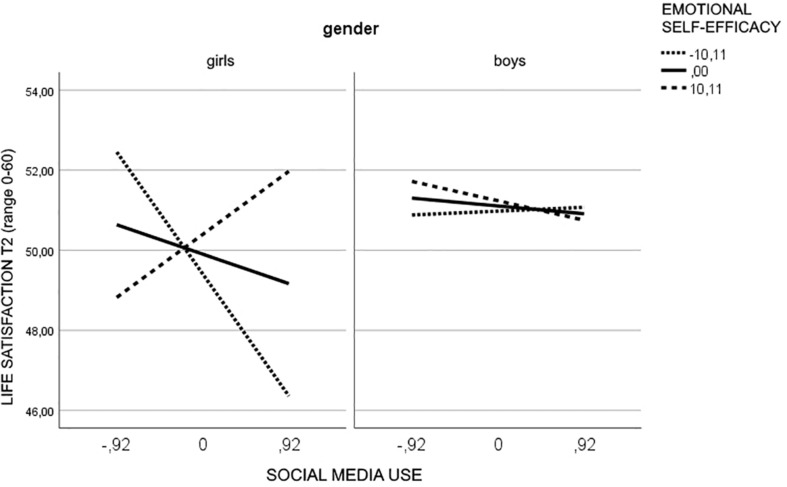
Moderating effect of emotional self-efficacy and gender in the relationship between social media use and life satisfaction.

The test of simple slopes showed that the interaction social media use × emotional self-efficacy was significant only for girls (*b* = 0.25, *p* = 0.0001). In particular, for girls with low levels of emotional self-efficacy high social media use was related to lower life satisfaction (*b* = −3.30, *t* = −4.65, *p* = 0.0001, 95% CI = −4.69, −1.90) whereas for girls with high levels of emotional self-efficacy high social media use was related to higher life satisfaction (*b* = 1.70, *t* = 2.07, *p* = 0.039, 95% CI = 0.09–3.32) (Figure 4).

## Discussion

This study focused on a very important period of life, generally considered one of the most delicate and challenging transitional periods in the life cycle. Between the ages of 12 and 14, boys and girls experience many of the pubertal changes, desire to spend more time with peers and also experience more dramatic mood swings than they did when they were children (Olivari and Confalonieri, 2013; Santrock, 2019). Moreover, today’s early adolescents are digital natives and the use of the Internet is part of their growing up experiences. Most of their daily activities, including friendships, involve the use of a device connected to the Internet.

It is in the interest of many researchers to understand the positive or negative effects of media use in adolescence. Our study contributed to this knowledge by investigating positive and negative outcomes of social media use from a complexity perspective, paying attention, in a longitudinal research design, to the possible role of some personal characteristics, such as gender and emotional self-efficacy.

From a descriptive point of view, in this study we observed that girls, compared to boys, spend more time on social media, report greater depressive feelings, lower affective well-being and lower life satisfaction. These results are in line with previous studies (Bandura et al., 2003; Pujazon-Zazik and Park, 2010; McLaughlin and King, 2015; Liang et al., 2016; González-Carrasco et al., 2017). The interpretation of such descriptive data can be traced back to the gender differences linked to the specific period of development considered. In fact, from early adolescence onward, girls attribute increasing importance to friendship, which they nurture also through increased use of social media. Moreover, they are usually more involved in internalized risk, which is expressed through increased feelings of depression, decreased well-being and decreased satisfaction with life.

With regard to the second and third aims, it was found that the use of social media does not in itself predict negative outcomes (depressive symptoms) or positive outcomes (affective well-being and life satisfaction). The effect of social media use on outcomes is moderated by the level of emotional self-efficacy, as we had assumed. Moreover, this moderating effect is in turn linked to gender, an aspect on which we had not made any assumptions. Notably, when girls possess high levels of self-efficacy in emotion regulation, greater social media use not only does not increase depressive feelings but is associated with greater feelings of affective well-being and higher life satisfaction. This finding is in line with other few studies that have emphasized positive social media outcomes in this period of life (Quinn and Oldmeadow, 2013; Ma, 2018). Conversely, when girls have low levels of emotional self-efficacy and use social media extensively, they experience more depressive feelings and lower affective well-being and life satisfaction. An explanation for these results can be found in the centrality of the emotional dimension for the development of early adolescent girls. As other studies have shown, there can be many difficulties in managing emotions in early adolescence and these usually lead to psychological distress and sometimes depression (Bandura et al., 2003; Grazzani et al., 2015; McLaughlin and King, 2015; Calandri et al., 2019, 2021). As mentioned above, early adolescent girls use the Internet mainly to maintain and nurture their friendships that are usually characterized by strong emotional charge. As some studies report, early adolescent girls prefer to talk about their feelings and emotions online than face-to-face and they use social media to escape from negative emotions more often than boys (HBSC (Health Behaviour in School-aged Children), 2018). These specific features could differentially expose girls to negative and positive developmental outcomes in relation to their level of emotional self-efficacy. Moreover, as Gardner and Davis (2013) describe, the emotional dimension is more difficult to control when communication does not take place face to face but is mediated by a device. Thus, early adolescents who are not yet equipped in their ability to manage their emotions but who use social media extensively are at greater risk with respect to depressive symptoms. Emotional self-efficacy is thus an indispensable skill for managing online friendships.

Our work complements the results of previous studies that associated social media use with negative or positive outcomes. The present work highlights a more complex relationship between social media use and outcomes. In early adolescent girls, the effects of social media use are moderated by an emotional skill, namely emotional self-efficacy.

Our study had some limitations. First of all, we only evaluated the amount of time spent daily on social media, without distinguishing between diverse social networking sites, nor between types of use. Social media are constantly and rapidly evolving and young people are likely to change their preferences over time (OFCOM, 2019). Each social networking site has specific characteristics and some research suggested that different social media have a specific impact on adolescents’ well-being (Phua et al., 2017). Moreover, it is important to differentiate between diverse types of social media use, especially between passive (e.g., scrolling, reading, and visiting profiles) and active (e.g., posting messages and/or pictures, interacting) (Ma, 2018; Orben et al., 2020; Schønning et al., 2020). The passive use is associated with lower well-being, probably because it stimulates mechanisms of social comparison and envy (Verduyn et al., 2017). Moreover, in our study the information about the amount of time spent daily on social media was based on participants’ self-report, whereas it would be advisable to integrate them with objective screen time measures. For example, adolescents could be asked to systematically self-record the time engaging in social media, or smartphone software that gives detailed reports about daily hours/minutes spent on diverse applications could be used. Self-report could underestimate or overestimate the actual amount of time spent on social media and this can affect the study results. Further research should therefore consider all these aspects when investigating social media use in relation to early adolescents’ subjective well-being.

Secondly, the small and not representative sample of our study did not allow us to generalize our results to the target population. Thirdly, a longitudinal design over a longer period of time could allow investigation of developmental trajectories and causal links between the variables considered.

Finally, in our study we did not find associations between social media use, emotional self-efficacy and outcomes for boys. These results suggest that other factors are likely to be associated with boys’ depressive symptoms and well-being during early adolescence. It is plausible that the non-significant results we found among boys are related to a different use of social networks between boys and girls that emerges from early adolescence onward. In turn, this different use is likely to be rooted in differences that more broadly characterize the friendships of boys from those of girls, especially during early adolescence. In particular, girls use social media more as a means of maintaining relationships and for self-expression, and tend to place more importance on social comparison (Bergagna and Tartaglia, 2018). Girls’ use of social media is therefore characterized by a high emotional involvement and our study suggests that the ability of managing emotions plays a crucial role with respect to the developmental outcomes. On the contrary, the use of social networks by boys is characterized by less emotional involvement, a reduced need to compare themselves with others and has a more playful dimension than for girls (Davis, 2012; Bergagna and Tartaglia, 2018). For this reason, the role of emotional self-efficacy might be less crucial for boys. Nonetheless, as gaming is more common among boys than girls and it has become as interactive as social media, future studies should examine the associations between online gaming, emotional self-efficacy and developmental outcomes, especially among boys.

The results of our work may offer useful indications for preventive interventions. Many countries are investing resources in digital education and it is important that this is guided by scientific data. In light of the results of this study, we can state that social media use in early adolescence can have important negative outcomes (in terms of increased depressive feelings), particularly among girls, but it can also have important positive outcomes (in terms of affective well-being and life satisfaction). This depends on the gender of the preadolescents and their personal skills, particularly with respect to managing emotions. Promoting emotional self-efficacy interventions can be very helpful in making the use of social media an opportunity for well-being and life satisfaction rather than a developmental risk. In fact, when girls possess a good level of emotional self-efficacy, the use of the Internet in social relationships becomes an additional resource. These girls experience greater feelings of well-being and enjoy greater satisfaction with the meaningful aspects of their lives, including social relationships.

## Data Availability Statement

The raw data supporting the conclusions of this article will be made available by the authors, without undue reservation, to any qualified researcher.

## Ethics Statement

The studies involving human participants were reviewed and approved by Comitato di Bioetica dell’Ateneo Università degli Studi di Torino. Written informed consent to participate in this study was provided by the participants’ legal guardian/next of kin.

## Author Contributions

EC conceived the study, provided statistical analysis and interpretation of the data, and wrote the manuscript. FG provided statistical analysis and interpretation of the data and wrote the manuscript. LR contributed to the interpretation of the results, collaborated in writing the discussion, and editing of the manuscript. All authors read and approved the final manuscript.

## Conflict of Interest

The authors declare that the research was conducted in the absence of any commercial or financial relationships that could be construed as a potential conflict of interest.

## Footnotes

1 In the Italian school system, there is a first cycle of education that lasts 5 years (primary school, from 6 to 11 years of age) and a second cycle that lasts 3 years (middle school, from 11 to 14 years of age).

2 Age distribution in the total sample:T1 *N* = 7 (12 years), *N* = 312 (13 years), *N* = 17 (14 years).T2 *N* = 7 (13 years), *N* = 312 (14 years), *N* = 17 (15 years). Mean age (*sd*) for girls and boys:T1 girls *M* = 13.01 (0.26); boys *M* = 13.05 (0.27).T2 girls *M* = 14.01 (0.26); boys *M* = 14.05 (0.27). Mean age did not differ between boys and girls, *t* = −1.56, *df* = 334, *p* = 0.120.

3 Family structure and parental levels of education were in line with Italian data (ISTAT, 2019).

4 No age differences were observed between girls and boys in the measured variables.

5 Measures were uncorrelated to age, except for a weak correlation between age and T1 depressive symptoms (*r* = 0.12, *p* = 0.03). The model predicting T2 depressive symptoms was tested also controlling for age, but this covariate was not statistically significant. Therefore, for parsimony the final model did not include age.
